# An analytical method for the identification of cell type-specific disease gene modules

**DOI:** 10.1186/s12967-020-02690-5

**Published:** 2021-01-06

**Authors:** Jinting Guan, Yiping Lin, Yang Wang, Junchao Gao, Guoli Ji

**Affiliations:** 1 Department of Automation, Xiamen University, Xiamen, China; 2 National Institute for Data Science in Health and Medicine, Xiamen University, Xiamen, China; 3 Department of Instrumental and Electrical Engineering, Xiamen University, Xiamen, China; 4 Innovation Center for Cell Signaling Network, Xiamen University, Xiamen, China

**Keywords:** Human brain, Cell type-specific, Gene network, Disease gene module

## Abstract

**Background:**

Genome-wide association studies have identified genetic variants associated with the risk of brain-related diseases, such as neurological and psychiatric disorders, while the causal variants and the specific vulnerable cell types are often needed to be studied. Many disease-associated genes are expressed in multiple cell types of human brains, while the pathologic variants affect primarily specific cell types. We hypothesize a model in which what determines the manifestation of a disease in a cell type is the presence of disease module comprised of disease-associated genes, instead of individual genes. Therefore, it is essential to identify the presence/absence of disease gene modules in cells.

**Methods:**

To characterize the cell type-specificity of brain-related diseases, we construct human brain cell type-specific gene interaction networks integrating human brain nucleus gene expression data with a referenced tissue-specific gene interaction network. Then from the cell type-specific gene interaction networks, we identify significant cell type-specific disease gene modules by performing statistical tests.

**Results:**

Between neurons and glia cells, the constructed cell type-specific gene networks and their gene functions are distinct. Then we identify cell type-specific disease gene modules associated with autism spectrum disorder and find that different gene modules are formed and distinct gene functions may be dysregulated in different cells. We also study the similarity and dissimilarity in cell type-specific disease gene modules among autism spectrum disorder, schizophrenia and bipolar disorder. The functions of neurons-specific disease gene modules are associated with synapse for all three diseases, while those in glia cells are different. To facilitate the use of our method, we develop an R package, CtsDGM, for the identification of cell type-specific disease gene modules.

**Conclusions:**

The results support our hypothesis that a disease manifests itself in a cell type through forming a statistically significant disease gene module. The identification of cell type-specific disease gene modules can promote the development of more targeted biomarkers and treatments for the disease. Our method can be applied for depicting the cell type heterogeneity of a given disease, and also for studying the similarity and dissimilarity between different disorders, providing new insights into the molecular mechanisms underlying the pathogenesis and progression of diseases.

## Background

In the past years, multiple tissue-specific referenced interactomes or gene interaction networks have been constructed [1–3], which promote to reveal the potential molecular mechanisms underlying human diseases. Studies have shown although many disease-associated genes are expressed in multiple tissues, the pathologic variants often affect primarily specific tissues [4–6]. It was hypothesized that what determines the manifestation of a disease in a tissue is the presence of disease gene module instead of individual genes [7]. A disease gene module is defined as a gene module comprised of disease-associated genes. Therefore, in addition to identifying individual disease-associated genes, it is also essential to identify the presence/absence of disease gene modules in tissues.

The advance of single-cell RNA sequencing (scRNA-seq) and single-nucleus RNA sequencing (snRNA-seq) have promoted the survey of cell atlases in heterogeneous tissues, such as human brains. The brain is a highly complex organ consisting of highly interconnected cells from different cell types. Although genome-wide association studies have identified genetic variants associated with the risk of brain-related diseases, such as neurological and psychiatric disorders, the causal variants and the specific cell types in which the disease-risk variants may be active are often needed to be studied. The transcriptional mechanisms controlling the developmental and functional properties of cell types in tissues from healthy and diseased individuals remain elusive [8]. Diverse cell types may be vulnerable for different brain-related disorders [9–13]. To identify the primary pathological cell types for a particular disease, especially for which the single-cell/nucleus RNA-seq data of diseased samples is not available, one kind of methods is to detect the cell type enrichments in susceptibility genes [13]. However, many disease-associated genes are expressed in multiple cell types, some of which do not show pathophysiological manifestations of the disease or of any functional abnormality. Therefore, here we further hypothesize that the presence of disease gene modules instead of individual genes determines the manifestation of a disease in cells from different cell types.

To characterize the cell type specificity of diseases in human brains, we first construct human brain cell type-specific gene interaction networks based on human brain nucleus gene expression data [14] and a referenced tissue-specific gene interaction network [1]. Then from the cell type-specific gene interaction networks, we identify candidate cell type-specific disease gene modules. By performing statistical tests, we assess the significance of the cell type-specific disease gene modules. Our analytical method can be applied for depicting the cell type heterogeneity of a given disease, and also for studying the similarity and dissimilarity between different diseases.

## Materials and methods

### Single nucleus gene expression data

We used the human brain nucleus gene expression data derived from middle temporal gyrus (MTG) of human cortex [14], which includes 15,928 nuclei originally sampled from eight human donor brains, of which 15,206 were from postmortem donors with no known neuropsychiatric or neurological conditions and 722 were from distal and normal tissues of neurosurgical donors. We downloaded the matrices of exon and intron read counts (the version of 2018) from Allen Institute for Brain Science and added them together to obtain gene expression data. Then we preprocessed the data with R packages of scater [15] and scran [16], including the quality control of nuclei and genes, and removing a minority of nuclei assigned to different cell cycle phases by the function of *cyclone* in scran. Nuclear and mitochondrial genes downloaded from Human MitoCarta2.0 [17] were excluded and protein-coding genes were retained. After removing the nuclei not assigned to any specific cell types, we obtained the final data matrix, which contains the expression level of 17,120 protein-coding genes in 12,246 nuclei, including 8994, 2762, 227, 3, 15, 112, and 133 nuclei from glutamatergic neuron (Gluta), GABAergic interneuron (GABA), astrocyte (Ast), endothelial (End), microglia (Mic), oligodendrocyte (Oli), and oligodendrocyte precursor cell (OPC), respectively.

### Tissue-specific gene interaction network

In order to depict the cell type specificity of genes in a tissue, we used the tissue-specific gene interaction network published in [1] as a referenced network. Because the human brain nucleus gene expression data we used was derived from MTG, a part of temporal lobe, we downloaded the temporal lobe-specific gene interaction network as a reference from the website of https://hb.flatironinstitute.org/download. The network only including the edges with evidence supporting a tissue-specific functional interaction (denoted as top edges) was used for the analyses.

### Construction of cell type-specific gene network

To identify cell type-specific gene interaction networks, we first calculated the counts per million (CPM) using the R package of edgeR [18]. Then we calculated cell type-specificity of genes, using a similar method in a study [19], which is defined as the minimum fold change in expression between the cell type of interest and each of the other cells. The specificity of gene $g$ in the interested cell type indexed by *c* is calculated as:

$${\text{specificity}}_{g,c} = \mathop {\hbox{min} }\limits_{{r \in \left[ {1,2, \ldots ,k} \right]\backslash c}} \frac{{\mathop \sum \nolimits_{i = 1}^{{N_{c} }} { \exp }\left( {i,g,c} \right)/N_{c} }}{{\mathop \sum \nolimits_{j = 1}^{{N_{r} }} { \exp }\left( {j,g,r} \right)/N_{r} }}$$

where each of *k* cell types is denoted by a numerical index from the set $\left( {1,2, \ldots ,k} \right)$, *r* denotes one cell type from the reference cell set, $N_{c}$ and $N_{r}$ are the numbers of nuclei classified into cell types *c* and *r* respectively, exp ($i,g,c$) denotes the expression of gene $g$ in nucleus *i* from cell type *c*. Next, to compare the cell type-specificity of a gene $g$ in a considered cell type *c* with those in other cell types, we calculated cell type score by comparing the cell type-specificity with the median and interquartile range (IQR) of its specificity across all cell types. The score of gene $g$ in the interested cell type *c* is calculated as:

$${\text{score}}_{g,c} = \frac{{{\text{specificity}}_{g,c} - median\left( {{\text{specificity}}_{g} } \right)}}{{IQR\left( {{\text{specificity}}_{g} } \right)}}$$

Then we extracted the genes with ${\text{score}}_{g,c}$ greater than a threshold in the considered cell type and the interactions between these genes from the referenced tissue-specific gene network, which is defined as cell type-specific gene interaction network. The threshold is recommended to be set as a positive value, which makes the cell type-specificity values of retained genes are larger than their medians across cell types and the retained genes would be more likely specific to the cell type.

### Disease-associated gene lists

To identify cell type-specific disease gene modules, we used gene lists associated with three kinds of neuropsychiatry diseases, autism spectrum disorder (ASD), schizophrenia (SCZ) and bipolar disorder (BPD). A total of 913 ASD candidate genes from Simons Foundation Autism Research Initiative (SFARI) were downloaded, which include 119, 144, 219, and 472 genes from categories S (syndromic), 1 (high confidence), 2 (strong candidate), and 3 (suggestive evidence). We downloaded genes associated with SCZ from SZDB [20], a database for schizophrenia genetic research, where these genes were identified by different kinds of studies including convergent functional genomics, CNV, differentially expression, GWAS, genetic linkage and association studies, *Sherlock* integrative analysis, and *Pascal* gene-based test. The genes supported by more than two kinds of studies, a total of 1419 genes, are used as SCZ-associated genes. We also downloaded 599 BPD candidate genes from BDgene database [21], each of which is positively supported by at least one kinds of studies.

### Identification of cell type-specific disease gene module

To identify cell type-specific gene module associated with a disease, we mapped the disease-associated genes onto the constructed cell type-specific gene interaction network, where the connected components among disease-associated genes were considered as candidate cell type-specific disease gene modules. We calculated the total number of disease-associated genes in the cell type-specific gene network (denoted as *T*), and the size of candidate disease gene module (denoted as *S*_*obs*_) which is the number of genes contained in the disease gene module. To access the significance of a candidate cell type-specific disease gene module, we performed permutation tests assuming that disease genes do not preferentially interact in the cell type-specific gene interaction network. With this null hypothesis, we selected *T* genes randomly in the cell type-specific gene interaction network and calculated the size of the largest connected component among these *T* genes, denoted as *S*_*rand*_. The procedure was repeated for 1000 times, and the *P*-value of permutations was determined by *n*/1000, where *n* is the number of largest connected components whose *S*_*rand*_ were greater than *S*_*obs*_ in the permutation tests. The correction for multiple testing was performed by controlling the false discovery rate (FDR) with the Benjamini–Hochberg method [22]. The candidate cell type-specific disease gene modules whose FDR-adjusted *P*-values < 0.1 are reported as significant.

## Results

**Fig. 1:**
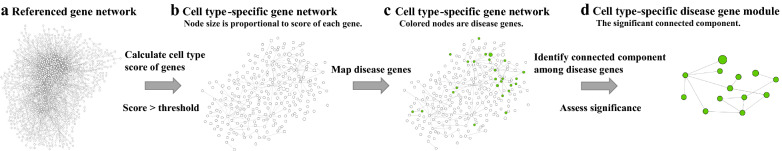
The analytical workflow. From **a** the referenced gene network, **b** a cell type-specific gene network is compiled by extracting genes with cell type scores greater than a threshold and their interactions. Then **c** disease genes are mapped onto the cell type-specific network and **d** the statistically significant cell type-specific disease gene module is identified by permutation tests

The analytical workflow can be seen in Fig. 1. To characterize the cell type-specificity of disease gene module, we first constructed cell type-specific gene interaction networks based on a referenced tissue-specific gene network. Specifically, for each cell type, we first calculated the cell type specificity of genes and obtained the cell type scores of genes (Materials and Methods), and then by extracting the genes with cell type scores greater than a threshold and their interactions from the referenced tissue-specific gene network (Fig. 1a), we constructed a cell type-specific gene interaction network (Fig. 1b). Next, we mapped the disease-associated genes onto each cell type-specific gene network (Fig. 1c) and the connected components among disease-associated genes were considered as candidate cell type-specific disease gene modules. By performing permutation tests (Materials and Methods), we identified statistically significant cell type-specific disease gene modules (Fig. 1d).

### Cell type-specific gene networks

Here we use the human brain nucleus gene expression data derived from middle temporal gyrus (MTG) of human cortex [14] and the temporal lobe-specific gene network as a referenced tissue-specific gene network [1] (Materials and Methods). After pre-processing, the MTG gene expression data includes 17,120 protein-coding genes. The temporal lobe-specific gene network contains 92,396,363 interactions between 25,825 genes. For enhancing reliability, we first filtered out the interactions whose weight values rank the last 20%, then the referenced gene network contains 1,289,258 interactions between 15,850 genes. After retaining the overlapping genes between genes in the referenced gene network and our analyzed gene expression data, the referenced gene network contained 1,042,968 interactions between 13,850 genes.

**Fig. 2:**
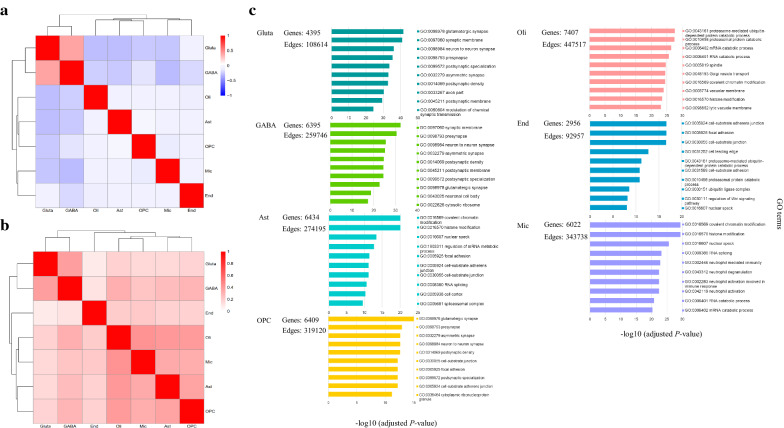
**a** The spearman correlation of gene scores between each pair of cell types. **b** Jaccard similarity between genes from each pair of cell type-specific gene networks. **c** For each cell type-specific gene network, the number of genes and interactions, and the top ten enriched GO terms are shown. Each color denotes each cell type

For each cell type, we first calculated the cell type specificity of genes and then calculated the cell type scores of genes. Additional file 1 lists the calculated cell type scores of genes whose cell type specificity are not “NA”, which are also used for the subsequent analyses. To examine the difference of gene scores between cell types, we computed the spearman correlation of gene scores between each pair of cell types (Fig. 2a). It can be seen that the cell type scores of genes in neurons are obviously distinct from those in glia cells, even showing a negative correlation, and there is almost no correlation among glia cells. These imply that the calculated cell type specificity of genes are different among different cell types.

Next, for each cell type, we compiled a cell type-specific gene network by extracting cell type-specific genes (genes with cell type scores > 0) and their interactions from the referenced tissue-specific gene network. To check the overlap of cell type-specific genes between cell types, we calculated Jaccard similarity between genes from each pair of cell type-specific gene networks (Fig. 2b), which also shows the distinction of cell type-specific genes between neurons and glia cells. At this threshold, on average 41.28% of genes were retained in a cell type, ranging from 21.34% for End to 53.48% for Oli; on average 25.28% of gene interactions were retained in a cell type, ranging from 8.91% for End to 42.91% for Oli (Fig. 2c). To characterize the gene functions of cell type-specific gene networks, we applied gene ontology analysis using clusterProfiler [23]. The GO term whose FDR-adjusted *P*-value < 0.1 and number of genes in the term is not less than ten was considered as significant (Additional file 2). The top ten enriched GO terms are shown for each cell type-specific gene network in Fig. 2c. For the two kinds of neurons, glutamatergic neuron (Gluta) and GABAergic interneuron (GABA), the cell type-specific gene networks are associated with the functioning of synapses, such as the functions of glutamatergic synapse, synaptic membrane, neuron to neuron synapse, and so on. For the glia cells, the enriched GO terms in the cell type-specific gene networks include covalent chromatin modification, histone modification, proteasomal protein catabolic process, mRNA catabolic process, cell-substrate junction, focal adhesion, neutrophil mediated immunity, neutrophil degranulation, and neutrophil activation. It can be seen that different kinds of gene functions are demonstrated in different cell types, especially between neurons and glia cells.

### Cell type-specificity of disease gene modules for a given disease

**Fig. 3:**
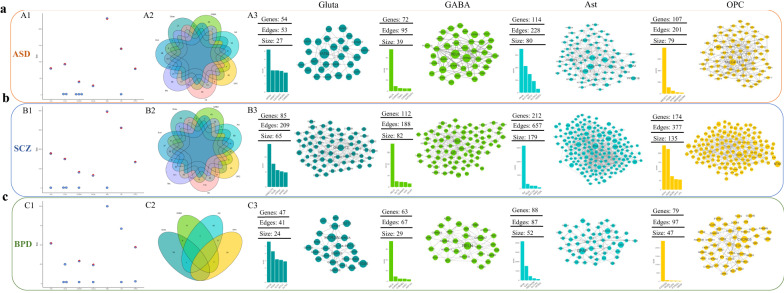
For **a** ASD, **b** SCZ and **c** BPD, the sizes of candidate cell type-specific disease gene modules and the identified significant disease modules (marked with *) are shown in A1, B1 and C1. A2, B2 and C2 are Venn diagrams of cell type-specific disease gene modules. A3, B3 and C3 show the genes and their interactions in the cell type-specific disease gene modules for Gluta, GABA, Ast and OPC. The numbers of disease-associated genes and their edges in the cell type-specific gene network, and the size of identified cell type-specific disease gene module are listed. The genes with top five cell type scores are also shown as bar plots

To illustrate how a disease manifests itself in particular cell types, we further identified cell type-specific disease gene modules. We first applied our analytical workflow for autism spectrum disorder (ASD), which is a set of neuropsychiatric disorders, characterized by impairments in social interaction and communication, and repetitive and restricted behaviors. We downloaded ASD-associated genes from SFARI and mapped the ASD genes onto each constructed cell type-specific gene network. The connected components among ASD genes in the cell type-specific gene network are regarded as candidate cell type-specific ASD gene modules. By performing permutation tests for 1000 times, we identified statistically significant cell type-specific ASD gene modules (Additional file 3: Figure S1A). In addition, we used stricter thresholds (score > 1 and score > 2) for constructing cell type-specific ASD gene modules (Additional file 3: Figure S1B, C). When using a stricter threshold, the obtained cell type-specific gene network and the resulting disease gene module would be a subset of the ones obtained using a less rigid threshold. If one would like to prioritize a disease gene module consisting of less genes, a stricter threshold should be used. In the rest of the article, for a clearer illustration of disease gene modules and for locating moderate number of genes, we report the cell type-specific ASD gene modules obtained using score threshold of one (Additional file 3: Figure S1B). For each cell type, Fig. 3A1 shows the sizes of candidate cell type-specific ASD gene modules, and the sizes of identified significant ASD gene modules. It can be seen that only the largest ASD gene module could be identified as significant. To examine the overlap between each pair of cell type-specific ASD gene modules, a Venn plot is shown as Fig. 3A2. Different gene modules are formed in different cells. In cell types of Gluta, GABA, Ast and OPC, the cell type-specific ASD gene modules have less overlap with others, and we also plotted the gene modules in Fig. 3A3. For each cell type-specific ASD gene module, we performed GO analysis and listed the included genes, their cell type scores and SFARI categories in Additional file 4. The genes with top five cell type scores in each cell type-specific ASD gene module are also shown in Additional file 5: Figure S2.

Table 1 lists the top five enriched GO terms for each cell type-specific ASD gene module. The functions of ASD gene modules are different among different cell types. For Gluta and GABA, the cell type-specific ASD gene modules are obviously associated with the functioning of synapses, while its dysregulation has been known to be involved in the development of ASD [24]. In brains, glutamate and GABA (gamma-aminobutyric acid) are major excitatory and inhibitory neurotransmitters, which pass messages at synapses from the presynaptic neuron to the postsynaptic neuron. For Gluta, gene *SATB2* with the largest cell type score is of note (Fig. 3A3), and *SATB2* belongs to categories of S and 3 in SFARI. For GABA, the cell type score of *RELN* is significantly larger than those of other genes (Fig. 3A3), and *RELN* belongs to category 1 in SFARI. Gene *RELN* encodes an extracellular matrix glycoprotein that is mostly synthesized in GABAergic interneurons in adulthood [25]. As to *SATB2* and *RELN*, their associations with ASD have been shown in previous studies [26–29]. The Ast-specific ASD gene module is related to functions of synapse organization and regulation of neuron projection development. Actually, astrocytes are integral partners with neurons in regulating synapse formation, development, organization, function and elimination [30, 31]. *PAX6* and *SLC1A2* are the top two genes in the Ast-specific ASD gene module (Fig. 3A3), and they are both syndromic genes. As to End, endothelial cells are involved in many aspects of vessel function, including formation of new blood vessels, which is called angiogenesis [32, 33], our identified End-specific ASD gene module are associated with regulations of angiogenesis and vasculature development. The gene with largest cell type score in the End-specific ASD gene module is *USP7* belonging to categories of S and 2 in SFARI (Additional file 5: Figure S2). The Mic-specific ASD gene module is related to the function of peptidyl-lysine modification. In Oli, the cell type-specific ASD gene module is involved with functions of regulation of cell morphogenesis, regulation of cell morphogenesis involved in differentiation, and regulation of transmembrane transporter activity. It can be seen that different gene modules are formed and different gene functions may be affected in different cell types by ASD. Our method has been shown to be effective in discovering cell type-specific disease-associated gene expression patterns.

**Table 1:** The top five enriched GO terms for cell type-specific disease gene modules

Cell type	ASD	SCZ	BPD
Ast	GO: 0098793 presynapse	GO: 0016358 dendrite development	GO: 0032922 circadian regulation of gene expression
	GO: 0050808 synapse organization	GO: 0050769 positive regulation of neurogenesis	GO: 0007623 circadian rhythm
	GO: 0010975 regulation of neuron projection development	GO: 0007409 axonogenesis	GO: 0048511 rhythmic process
	/	GO: 0010975 regulation of neuron projection development	GO: 0042752 regulation of circadian rhythm
	/	GO: 1901214 regulation of neuron death	GO: 0050795 regulation of behavior
GABA	GO: 0060078 regulation of postsynaptic membrane potential	GO: 0098982 GABA-ergic synapse	GO: 1902495 transmembrane transporter complex
	GO: 0042391 regulation of membrane potential	GO: 0098793 presynapse	GO: 1990351 transporter complex
	GO: 0045211 postsynaptic membrane	GO: 0097060 synaptic membrane	GO: 0022824 transmitter-gated ion channel activity
	GO: 1902495 transmembrane transporter complex	GO: 0045211 postsynaptic membrane	GO: 0022835 transmitter-gated channel activity
	GO: 1990351 transporter complex	GO: 0060078 regulation of postsynaptic membrane potential	GO: 0005230 extracellular ligand-gated ion channel activity
Gluta	GO: 0097060 synaptic membrane	GO: 0097060 synaptic membrane	GO: 0097060 synaptic membrane
	GO: 0045211 postsynaptic membrane	GO: 0045211 postsynaptic membrane	GO: 0045211 postsynaptic membrane
	GO: 0099572 postsynaptic specialization	GO: 0099572 postsynaptic specialization	GO: 0034702 ion channel complex
	GO: 0098984 neuron to neuron synapse	GO: 0098978 glutamatergic synapse	GO: 1902495 transmembrane transporter complex
	GO: 0022839 ion gated channel activity	GO: 0098984 neuron to neuron synapse	GO: 1990351 transporter complex
OPC	GO: 0097060 synaptic membrane	GO: 0098978 glutamatergic synapse	GO: 0045211 postsynaptic membrane
	GO: 0045211 postsynaptic membrane	GO: 0051961 negative regulation of nervous system development	GO: 0097060 synaptic membrane
	GO: 0099572 postsynaptic specialization	GO: 0032279 asymmetric synapse	GO: 0034702 ion channel complex
	GO: 0098984 neuron to neuron synapse	GO: 0098984 neuron to neuron synapse	GO: 1902495 transmembrane transporter complex
	GO: 0032279 asymmetric synapse	GO: 0097060 synaptic membrane	GO: 0032279 asymmetric synapse
End	GO: 0016570 histone modification	GO: 0030902 hindbrain development	/
	GO: 0016569 covalent chromatin modification	GO: 0005925 focal adhesion	
	GO: 0045765 regulation of angiogenesis	GO: 0005924 cell-substrate adherens junction	
	GO: 0033044 regulation of chromosome organization	GO: 0030055 cell-substrate junction	
	GO: 1901342 regulation of vasculature development	GO: 1903706 regulation of hemopoiesis	
Mic	GO: 0016569 covalent chromatin modification	GO: 0050769 positive regulation of neurogenesis	/
	GO: 0016570 histone modification	GO: 0010975 regulation of neuron projection development	
	GO: 0018205 peptidyl-lysine modification	GO: 0016049 cell growth	
	GO: 0031056 regulation of histone modification	GO: 0031346 positive regulation of cell projection organization	
	GO: 1902275 regulation of chromatin organization	GO: 0070997 neuron death	
Oli	GO: 0016569 covalent chromatin modification	GO: 0010975 regulation of neuron projection development	/
	GO: 0022604 regulation of cell morphogenesis	GO: 0043025 neuronal cell body	
	GO: 0010769 regulation of cell morphogenesis involved in differentiation	GO: 0007409 axonogenesis	
	GO: 0016570 histone modification	GO: 0098793 presynapse	
	GO: 0022898 regulation of transmembrane transporter activity	GO: 0050769 positive regulation of neurogenesis	

As to the methods for identifying cell type-specific disease-associated gene modules, the most straightforward way is using clustering algorithm to first detect gene modules from cell type-specific gene interaction network, and then identifying gene modules enriched with disease-related genes. Therefore, we also applied Louvain clustering by using R package of igraph [34] to identify cell type-specific gene modules. Then we detected cell type-specific gene modules enriched with SFARI ASD genes. We found that in most of cell types, there is only one gene module which is enriched with ASD genes and contains more than five genes, no matter using score threshold of zero or one (Additional file 6: Figure S3A1, B1). For these ASD gene-enriched modules, we checked if they are significantly overlapping with our identified disease gene modules. It is found that our disease gene module significantly overlaps with the ASD gene-enriched module/modules and in most cell types, only overlaps with one ASD gene-enriched module (Additional file 6: Figure S3A2, B2). Therefore, it has been proven that our method is effective and the results are consistent with the ones obtained using clustering methods.

The example application of our analytical method to ASD supports our hypothesis that a disease manifests itself in a cell type through forming a statistically significant disease gene module. It is essential to detect and compare the cell type-specific disease gene modules for studying the cell type heterogeneity of a given disease. The identification of cell type-specific disease gene modules can promote the development of more targeted biomarkers and treatments for the disease.

### Similarity and dissimilarity of cell type-specific disease gene modules between diseases

In addition to characterizing the cell type heterogeneity of a given disease, our analytical pipeline can be applied to study the similarity and dissimilarity of cell type-specific disease gene modules between diseases. Schizophrenia (SCZ) and bipolar disorder (BPD) are two kinds of neuropsychiatry disorders sharing similar clinical manifestations with ASD, suggesting shared genetic influences and common biological mechanisms underlying these disorders. To study the effect of genetic correlation in these disorders and illustrate the similarity and difference between disease gene modules in a given cell type, we also identified cell type-specific SCZ (Additional file 7: Figure S4, Fig. 3b) and BPD (Fig. 3c) gene modules using cell type score threshold of one. From Fig. 3B1, C1, it also can be seen that only the largest candidate SCZ/BPD gene module could be identified as significant. For each significant cell type-specific SCZ or BPD gene module, we listed the enriched GO terms, the included genes, their cell type scores and SZDB or BDgene database categories in Additional files 8 and 9. The genes with top five cell type scores in the cell type-specific SCZ gene modules are also shown in Additional file 5: Figure S2. For BPD, only in four cell types, Gluta, GABA, Ast and OPC, cell type-specific BPD gene modules were identified as significant (Fig. 3C1). Therefore, for comparison, Fig. 3A3, B3, and C3 show the cell type-specific ASD, SCZ and BPD gene modules in these four cell types along with the genes with top five cell type scores.

Table 1 also lists the top five enriched GO terms for each cell type-specific SCZ and BPD gene modules. In the two kinds of neurons, the functions of cell type-specific disease gene modules for all three diseases are associated with synapses, while in the glia cells, those are different among ASD, SCZ and BPD. For instance, in Ast, the cell type-specific ASD gene module is associated with the functions of presynapse, synapse organization and regulation of neuron projection development, and the SCZ module is associated with dendrite development, positive regulation of neurogenesis, axonogenesis, regulations of neuron projection development and neuron death, while the BPD module is related to circadian rhythm and regulation of behavior. This demonstrates that the contributing disease-associated genes in astrocytes are different in BPD compared with ASD and SCZ. For End, ASD gene module is involved with functions of histone modification, covalent chromatin modification, regulation of angiogenesis, and regulation of vasculature development, while SCZ gene module is related to functions of hindbrain development, focal adhesion, cell-substrate junction, and regulation of hemopoiesis. For Oli-specific ASD gene module, its gene functions include covalent chromatin modification, regulation of cell morphogenesis, histone modification and regulation of transmembrane transporter activity, while the SCZ gene module is associated with regulation of neuron projection development, neuronal cell body, axonogenesis, and positive regulation of neurogenesis. From the perspective of disease gene modules, it can be noted that different gene modules are identified and the genes with top cell type scores are distinct between different diseases (Fig. 3c, Additional file 5: Figure S2), while it is interesting that gene *RELN* has the largest cell type score in GABA-specific disease gene modules for all three diseases. Gene *RELN* is essential in synaptic plasticity, dendritic morphology, and cognitive function [25]. Several studies have shown the role of *RELN* in the susceptibility to ASD [29, 35], SCZ [25, 36] and BPD [37].

### CtsDGM: an R package for identifying cell type-specific disease gene module

**Fig. 4:**
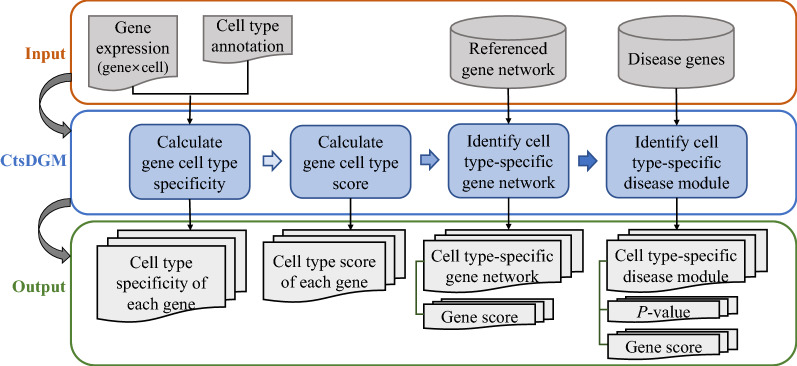
The input, output and workflow of CtsDGM

To facilitate the use of our analytical workflow, we developed an R package, CtsDGM, for the identification of cell type-specific disease gene modules. CtsDGM contains four components, including the calculation of cell type specificity and scores of genes, the identification of cell type-specific gene networks and cell type-specific disease gene modules (Fig. 4). The input data include a gene expression matrix (row: gene, column: cell) with row and column names, a list of cell type annotation with each denoting each cell in the gene expression matrix, a referenced gene interaction network with each row recording the gene–gene pair, and a list of disease-associated genes. CtsDGM can calculate cell type specificity and cell type score of each gene in each cell type using the gene expression matrix and the cell type annotation. Next, by setting a cell type score threshold, cell type-specific gene interaction networks can be obtained from the referenced gene interaction network along with gene cell type scores. Then using a list of disease-associated genes, CtsDGM can identify significant cell type-specific disease gene modules by performing permutation tests, and output the modules, FDR-corrected permutation *P*-values, and gene cell type scores. CtsDGM is available at https://github.com/JGuan-lab/CtsDGM.

CtsDGM can be used according to the need of users. One can just use CtsDGM to calculate gene cell type specificity or score, or identify cell type-specific gene interaction network. About defining cell type-specific gene interaction network, our package is flexible to allow ones to input gene scores calculated by other methods, such as applying the tissue-specificity metrics reviewed in a study [38] to scRNA-seq/snRNA-seq data, and then CtsDGM can extract cell type-specific gene network by setting a threshold. The necessary input data for our analytical method has been readily accessible, which makes our method applicable for other diseases. There have been several studies providing tissue-specific interactomes or gene interaction networks. For instance, the temporal lobe-specific gene interaction network we used in this study was downloaded from GIANT at the website of https://hb.flatironinstitute.org/download where many other tissue-specific gene interaction networks are also provided [1]. Besides, a recent study has evaluated the existing gene interaction networks, and also created a parsimonious composite network (PCNet) with both high efficiency and performance [2]. PCNet and other evaluated gene interaction networks are integrated and deposited on NDEx with UUID: f93f402c-86d4-11e7-a10d-0ac135e8bacf. In addition, there have been more and more public scRNA-seq or snRNA-seq datasets. For example, Allen Institute for Brain Science provides human or mouse brain-related datasets, and Hemberg's group at the Sanger Institute provides a collection of publicly available datasets, such as the ones involving human brain, liver and pancreas, mouse brain, pancreas, and retina, which can be accessed at https://hemberg-lab.github.io/scRNA.seq.datasets/. One can choose the dataset to use according to the disease focused on.

## Discussion

The advance of scRNA-seq and snRNA-seq have promoted the survey of cell type heterogeneity in human brains. Genome-wide association studies have identified genetic variants associated with the risk of brain-related diseases, while the causal variants and the specific vulnerable cell types are often needed to be studied. To identify the primary pathological cell types for a particular disease, especially for which the single-cell/nucleus RNA-seq data of diseased samples is not available, one kind of methods is to detect the cell type enrichments in susceptibility genes. However, many disease-associated genes are expressed in multiple cell types, the pathologic variants affect primarily specific cells while other cells do not show pathophysiological manifestations of the disease or of any functional abnormality. Therefore, we hypothesize that the presence of disease gene modules instead of individual genes determines the manifestation of a disease in cells.

To characterize the cell type specificity of brain-related diseases, we first constructed human brain cell type-specific gene interaction networks based on human brain nucleus gene expression data and a referenced tissue-specific gene interaction network. Then we mapped disease-associated genes onto the cell type-specific gene interaction networks and identified significant connected components among disease genes by performing statistical tests, which are defined as cell type-specific disease gene modules. First, we identified cell type-specific ASD gene modules for studying the cell type heterogeneity of ASD. We found that only the largest connected components among ASD genes could be identified as significant disease gene module. Different gene modules are formed in different cells, and distinct gene functions are demonstrated in different cell type-specific ASD gene modules. For instance, the Gluta- and GABA-specific ASD gene modules are involved with the functioning of synapses; the Ast-specific ASD gene module is associated with the functions of synapse organization and regulation of neuron projection development; the End-specific ASD gene module is associated with regulations of angiogenesis and vasculature development; the Oli-specific ASD gene module is related to regulation of cell morphogenesis. In addition, distinct genes demonstrate the top cell type scores in different cells, which implies the primary causal genes are different across cells.

As to the methods for identifying cell type-specific disease-associated gene modules, one may think of the most straightforward way that using clustering algorithm to first detect gene modules from cell type-specific gene interaction network, and then identifying the ones enriched with disease-related genes. Therefore, we also applied Louvain clustering to perform the analysis and identified cell type-specific gene modules enriched with known ASD genes. We found that only in few cell types, there are more than one module which are enriched with ASD genes and contain more than five genes. In addition, our identified cell type-specific disease gene module could overlap with the ASD gene-enriched module/modules, and in most cell types, only overlaps with one ASD gene-enriched module. Therefore, it has been proven that our method is effective and the results are consistent with the ones obtained using clustering methods.

Moreover, to study the influence of genetic overlap among ASD, SCZ and BPD, we study the similarity and dissimilarity among their cell type-specific disease gene modules. For the two kinds of neurons, the functions of cell type-specific disease gene modules are associated with synapse for all three diseases, while those are different in glia cells. For instance, in astrocytes, the cell type-specific ASD gene module is associated with the functions of presynapse, synapse organization and regulation of neuron projection development, and the SCZ module is associated with dendrite development, positive regulation of neurogenesis, axonogenesis, regulations of neuron projection development and neuron death, while the BPD module is related to circadian rhythm and regulation of behavior. For Oli-specific ASD gene module, its gene functions include covalent chromatin modification, regulation of cell morphogenesis, histone modification and regulation of transmembrane transporter activity, while the SCZ gene module is associated with regulation of neuron projection development, neuronal cell body, axonogenesis, and positive regulation of neurogenesis. From the perspective of disease gene modules, it can be noted that different gene modules are identified and the genes with top cell type scores are different between diseases.

Our method has been shown to be effective in discovering cell type-specific disease-associated gene expression patterns. The results support our hypothesis that a disease manifests itself in a cell type through forming a statistically significant disease gene module. The identification of cell type-specific disease gene modules can promote the development of more targeted biomarkers and treatments for the disease. Our analytical pipeline can be applied for depicting the cell type heterogeneity of a given disease and studying the similarity and dissimilarity between different diseases.

## Conclusion

We constructed cell type-specific gene interaction networks by integrating single nucleus gene expression data with a referenced gene network. Then statistically significant cell type-specific disease gene modules were identified by performing permutation tests. We also developed an R package to facilitate the use of our analytical pipeline. Our method can be applied for depicting the cell type heterogeneity of a given disease and studying the similarity and dissimilarity between different diseases, providing new insights into the molecular mechanisms underlying the pathogenesis and progression of diseases.

## Supplementary information

**Additional file 1.** The calculated cell type-specificity and scores of genes in each cell type.

**Additional file 2.** The enriched GO terms for cell type-specific gene networks obtained using cell type score threshold of zero.

**Additional file 3: Figure S1.** The identified cell type-specific ASD gene modules obtained using score threshold of (A) zero, (B) one and (C) two. The numbers of disease-associated genes and their edges in the cell type-specific gene network, and the size of identified cell type-specific disease gene module are listed.

**Additional file 4.** The genes, their cell type scores and SAFRI categories in the cell type-specific ASD gene modules obtained using cell type score threshold of one. The enriched GO terms are listed.

**Additional file 5: Figure S2.** The genes with top five cell type scores in each identified cell type-specific ASD and SCZ gene module obtained using score threshold of one.

**Additional file 6: Figure S3.** For cell type-specific gene interaction network obtained using score threshold of zero (A) and one (B), Louvain clustering was applied to identify gene modules. For these gene modules, the enrichment with ASD risk genes is assessed in A1 and B1. For the ASD gene-enriched modules containing more than five genes, the overlap between them and the identified cell type-specific disease gene module by our method was assessed in A2 and B2.

**Additional file 7: Figure S4.** The identified cell type-specific SCZ gene modules obtained using score threshold of one.

**Additional file 8.** The genes, their cell type scores and SZDB categories in the cell type-specific SCZ gene modules obtained using score threshold of one. The enriched GO terms are listed.

**Additional file 9.** The genes, their cell type scores and BDgene database categories in the cell type-specific BPD gene modules obtained using score threshold of one. The enriched GO terms are listed.

## Data Availability

The analyzed datasets and codes in this study are available at https://github.com/JGuan-lab/CtsDGM.

## Abbreviations

ASD: Autism spectrum disorder
SCZ: Schizophrenia
BPD: Bipolar disorder
MTG: Middle temporal gyrus
Gluta: Glutamatergic neuron
GABA: GABAergic interneuron
Ast: Astrocyte
End: Endothelial
Mic: Microglia
Oli: Oligodendrocyte
OPC: Oligodendrocyte precursor cell
