# Supplementary material for: An analytical method for the identification of cell type-specific disease gene modules
Source: J Transl Med. 2021 Jan 6;19:20. doi: 10.1186/s12967-020-02690-5 (PMC7788893; doi:10.1186/s12967-020-02690-5)

A. Cell type-specific ASD gene module (score>0)

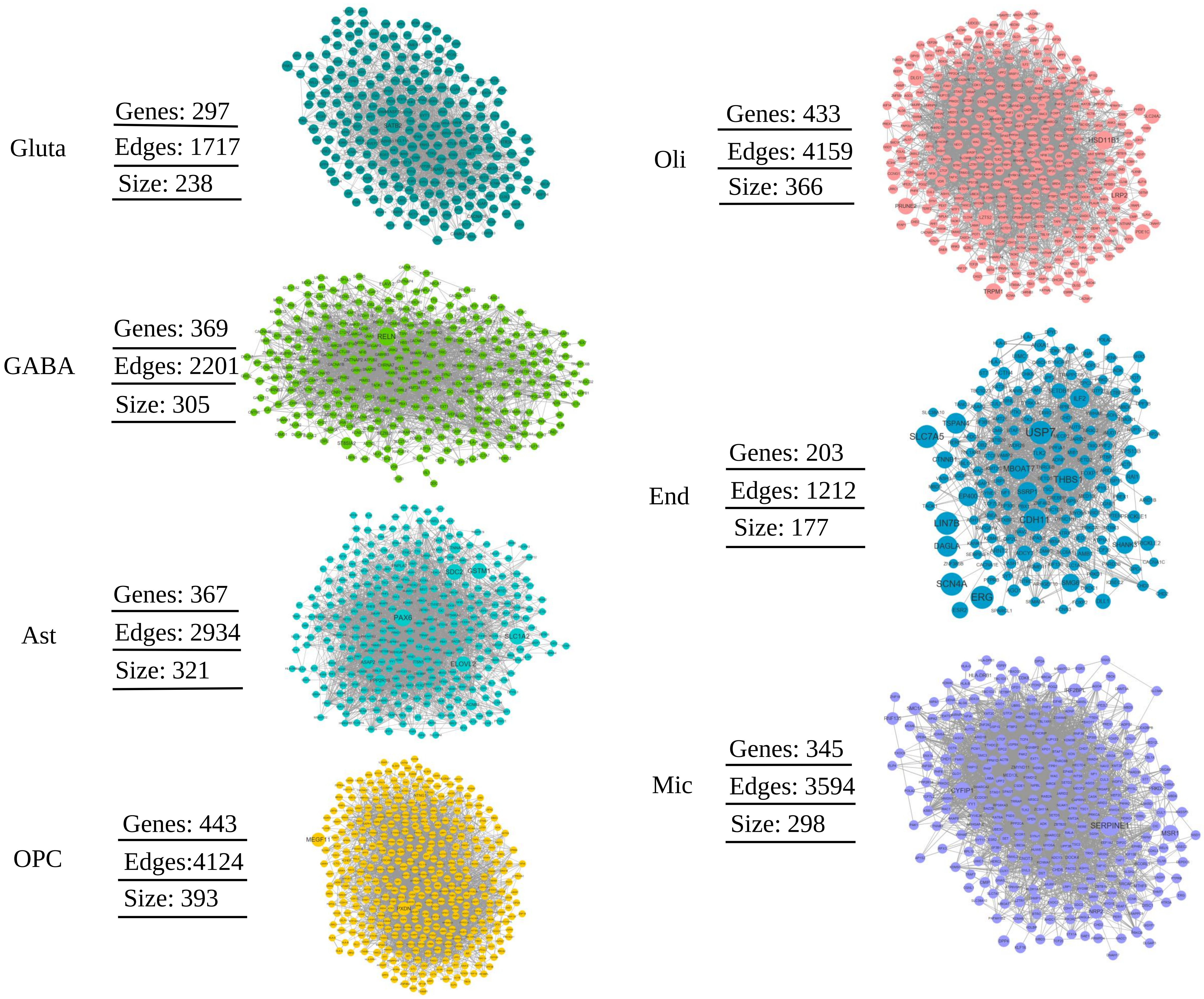

B. Cell type-specific ASD gene module (score>1)

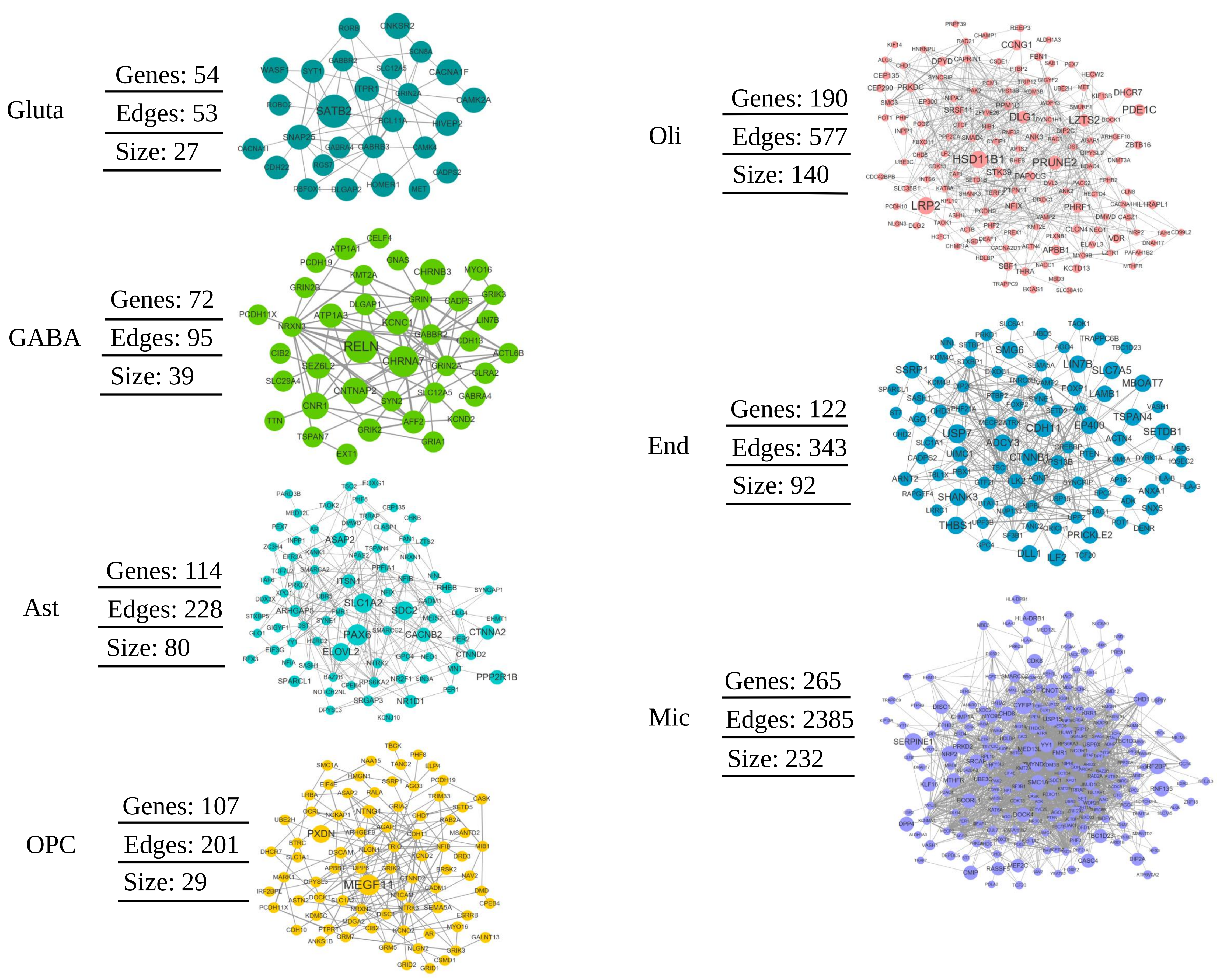

C. Cell type-specific ASD gene module (score>2)

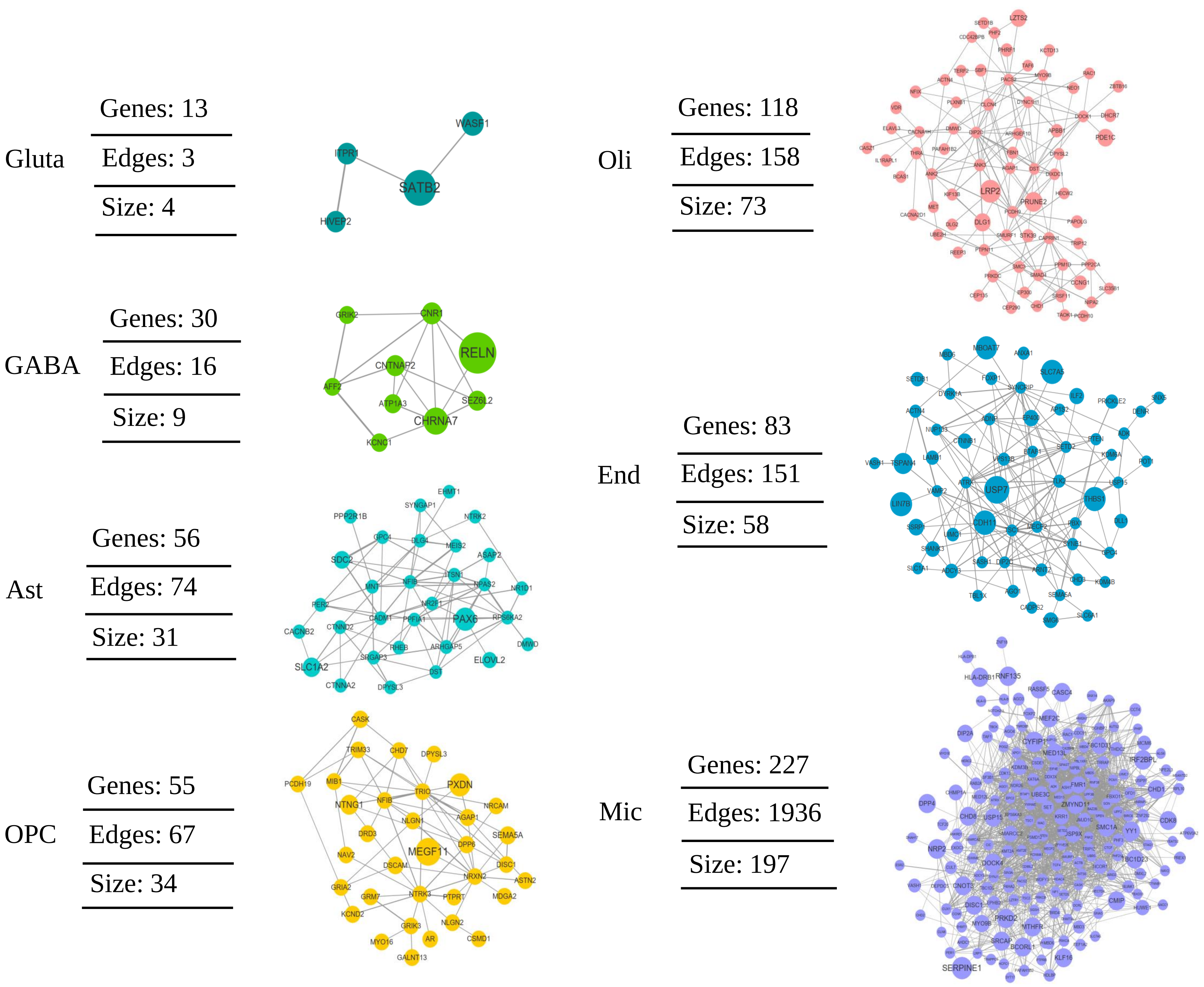

Supplement: Supplementary file 3 — Additional file 3: Figure S1. The identified cell type-specific ASD gene modules obtained using score threshold of (A) zero, (B) one and (C) two. The numbers of disease-associated genes and their edges in the cell type-specific gene network, and the size of identified cell type-specific disease gene module are listed. [file 12967_2020_2690_MOESM3_ESM.pdf]
