# Supplementary figures and images for: An analytical method for the identification of cell type-specific disease gene modules
Source: J Transl Med. 2021 Jan 6;19:20. doi: 10.1186/s12967-020-02690-5 (PMC7788893; doi:10.1186/s12967-020-02690-5)

ASD

Gluta

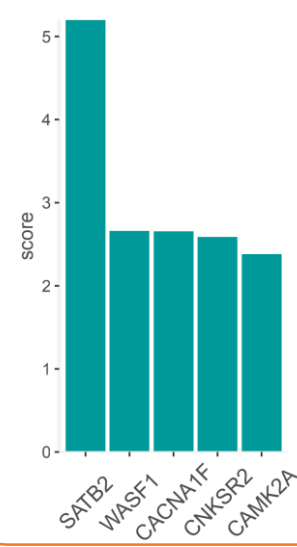

GABA

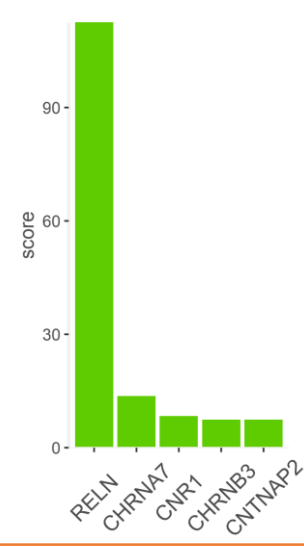

Ast

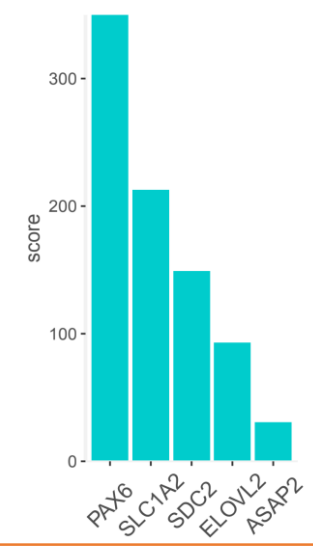

OPC

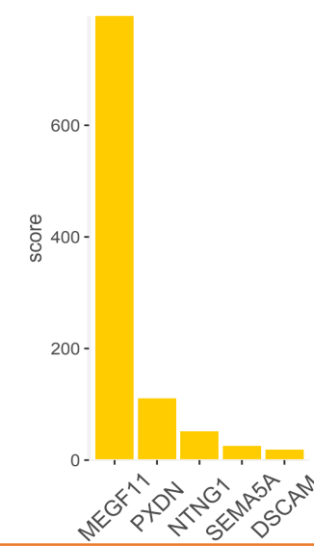

Oli

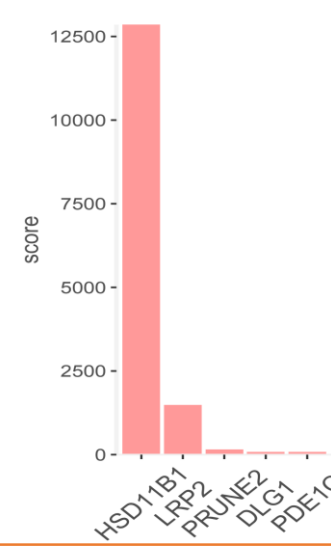

End

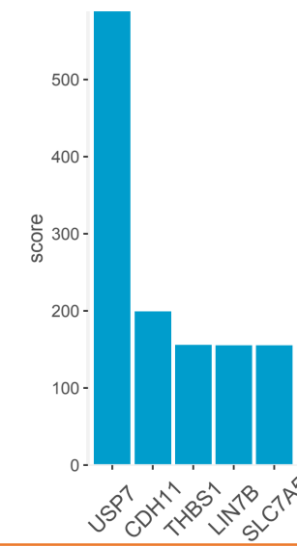

Mic

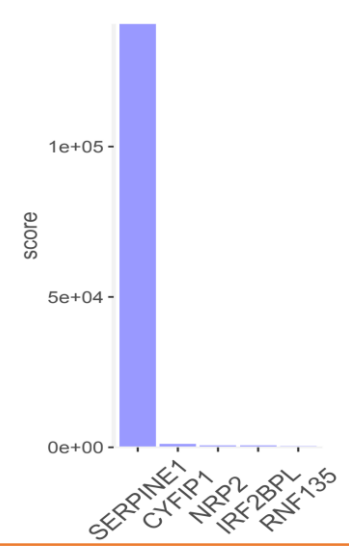

SCZ

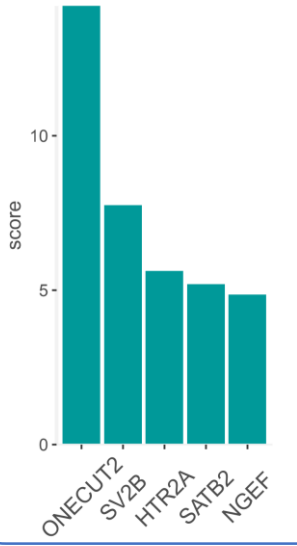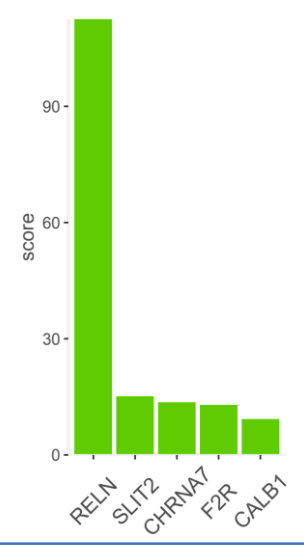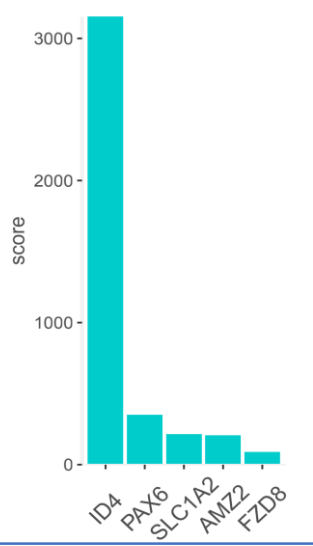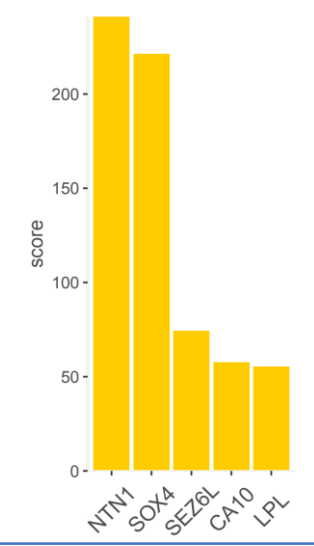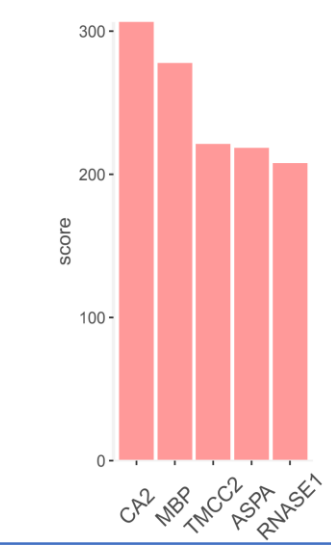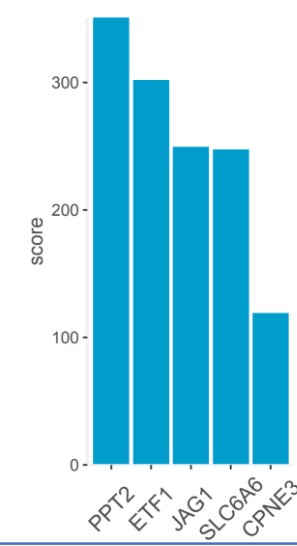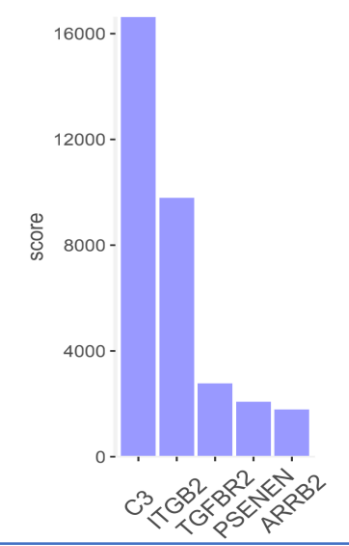

Supplement: Supplementary file 5 — Additional file 5: Figure S2. The genes with top five cell type scores in each identified cell type-specific ASD and SCZ gene module obtained using score threshold of one. [file 12967_2020_2690_MOESM5_ESM.pdf]

A1

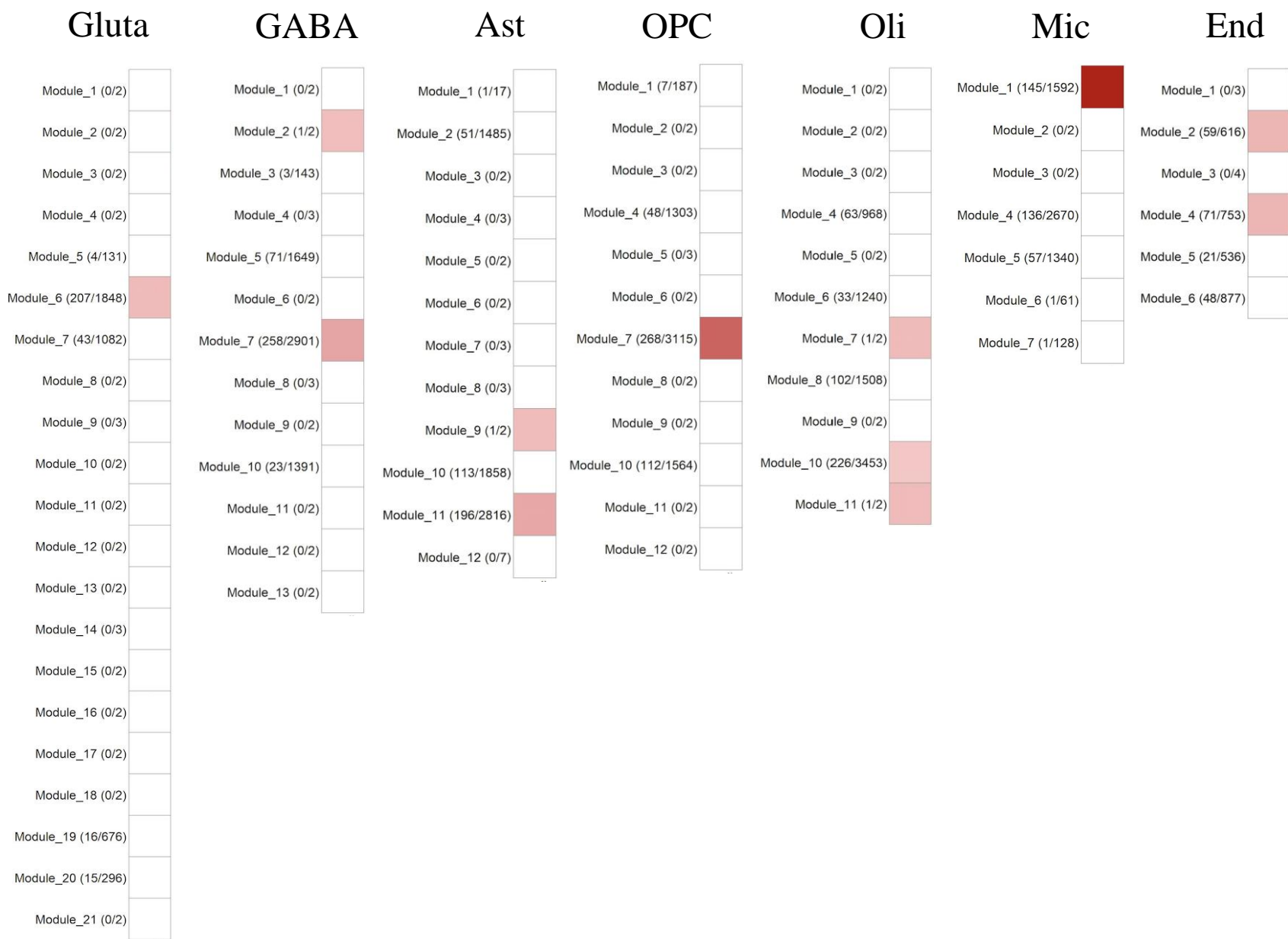

A2

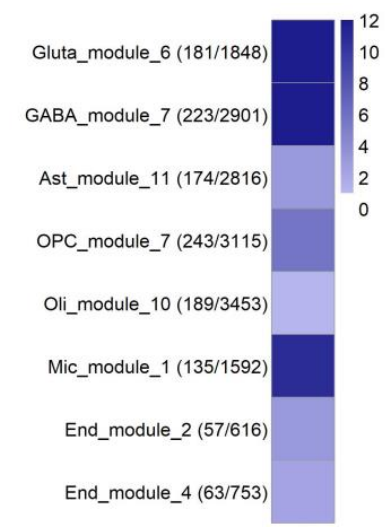

B1

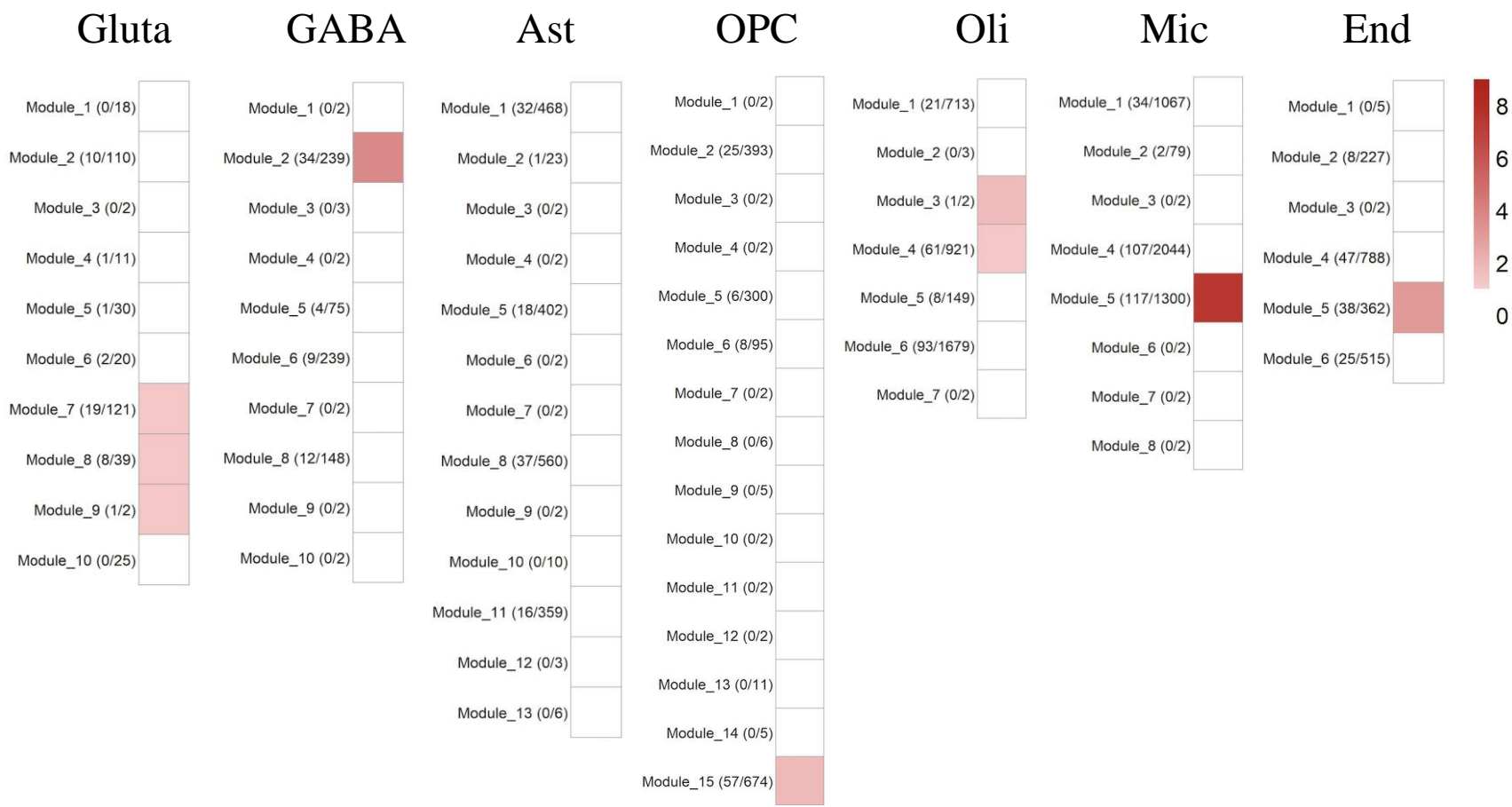

B2

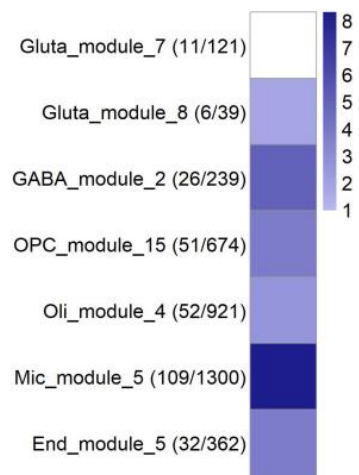

Supplement: Supplementary file 6 — Additional file 6: Figure S3. For cell type-specific gene interaction network obtained using score threshold of zero (A) and one (B), Louvain clustering was applied to identify gene modules. For these gene modules, the enrichment with ASD risk genes is assessed in A1 and B1. For the ASD gene-enriched modules containing more than five genes, the overlap between them and the identified cell type-specific disease gene module by our method was assessed in A2 and B2. [file 12967_2020_2690_MOESM6_ESM.pdf]
