# Supplementary material for: An analytical method for the identification of cell type-specific disease gene modules
Source: J Transl Med. 2021 Jan 6;19:20. doi: 10.1186/s12967-020-02690-5 (PMC7788893; doi:10.1186/s12967-020-02690-5)

Cell type-specific SCZ gene module (score>1)

Gluta

Genes: 85
Edges: 209
Size: 65

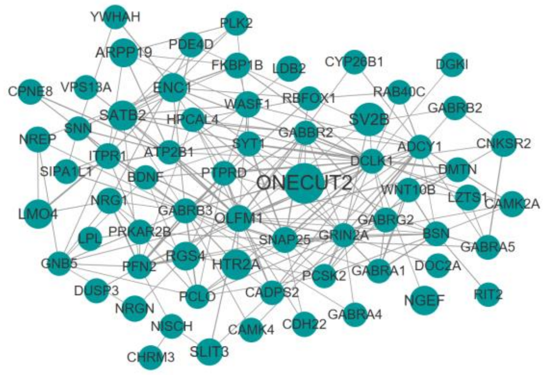

GABA

Genes: 112
Edges: 188
Size: 82

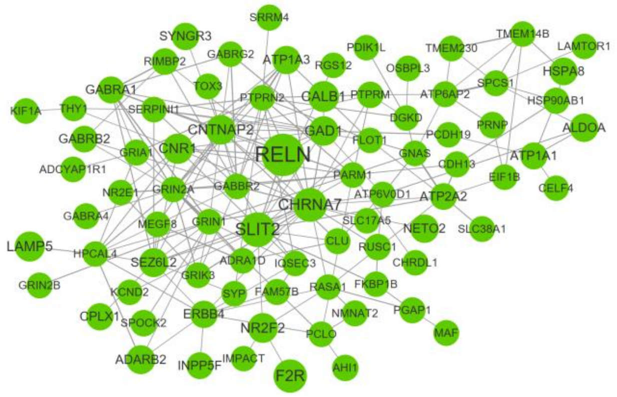

Ast

Genes: 212
Edges: 657
Size: 179

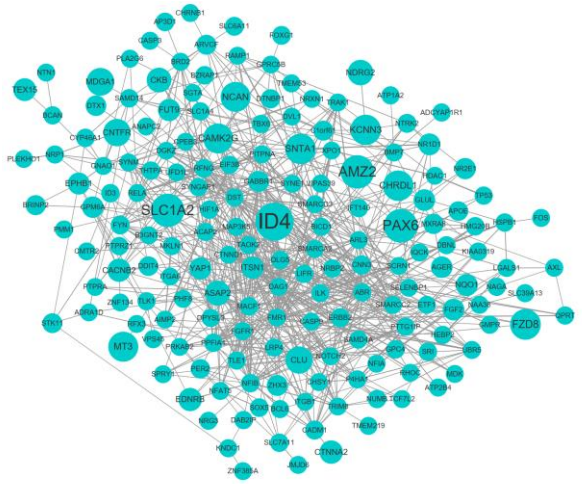

OPC

Genes: 174
Edges: 377
Size: 135

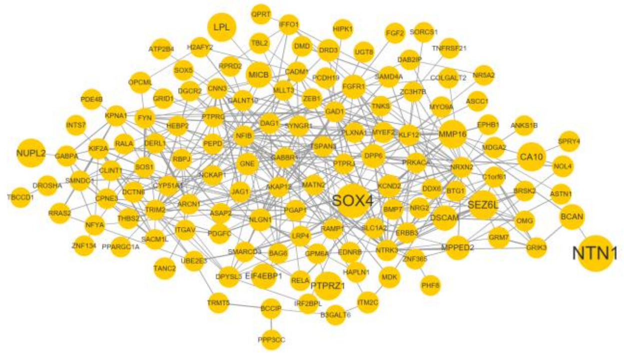

Oli

Genes: 354
Edges: 2147
Size: 311

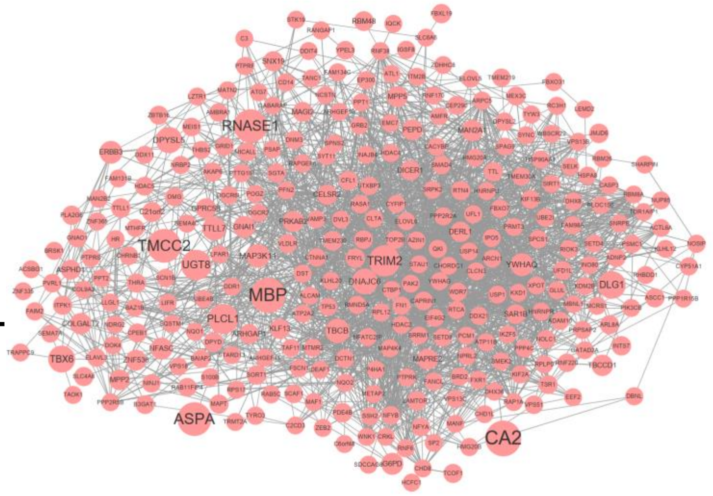

End

Genes: 184
Edges: 553
Size: 151

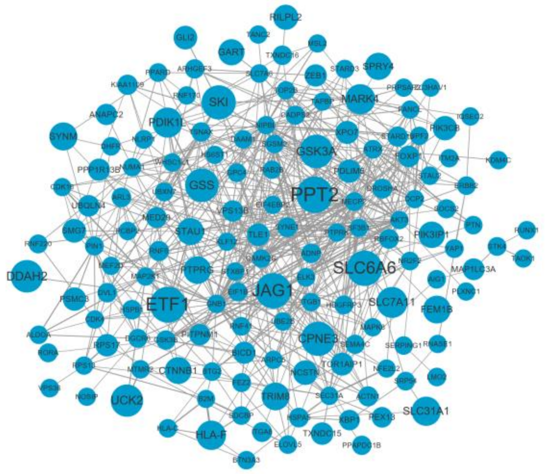

Mic

Genes: 431
Edges: 4269
Size: 296

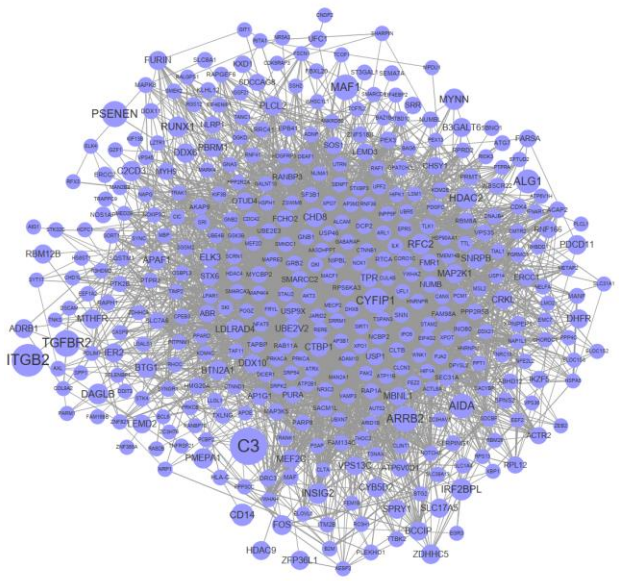

Supplement: Supplementary file 7 — Additional file 7: Figure S4. The identified cell type-specific SCZ gene modules obtained using score threshold of one. [file 12967_2020_2690_MOESM7_ESM.pdf]
